# Health Literacy and Internet Use Among Japanese Older Adults: A Gender-Stratified Cross-Sectional Analysis of the Moderating Effects of Neighborhood Relationships

**DOI:** 10.3390/healthcare13010056

**Published:** 2024-12-31

**Authors:** Tsubasa Nakada, Kayo Kurotani, Satoshi Seino, Takako Kozawa, Shinichi Murota, Miki Eto, Junko Shimasawa, Yumiko Shimizu, Shinobu Tsurugano, Fuminori Katsukawa, Kazunori Sakamoto, Hironori Washizaki, Yo Ishigaki, Maki Sakamoto, Keiki Takadama, Keiji Yanai, Osamu Matsuo, Chiyoko Kameue, Hitomi Suzuki, Kazunori Ohkawara

**Affiliations:** 1 Graduate School of Informatics and Engineering, The University of Electro-Communications, Tokyo 182-8585, Japan; tsubasanakada@uec.ac.jp (T.N.); maki.sakamoto@uec.ac.jp (M.S.); takadama@g.ecc.u-tokyo.ac.jp (K.T.); yanai@cs.uec.ac.jp (K.Y.); ma004017@edu.cc.uec.ac.jp (O.M.);; 2 Graduate School of Life Sciences, Showa Women’s University, Tokyo 154-8533, Japan; 3 Institute of Well-Being, Yamagata University, Yamagata 990-9585, Japan; seino.s@med.id.yamagata-u.ac.jp; 4 Faculty of Human Health, Komazawa Women’s University, Tokyo 206-8511, Japan; t-kozawa@komajo.ac.jp; 5 Faculty of Humanities and Social Sciences, Tokyo Metropolitan University, Tokyo 192-0397, Japan; shin1@tmu.ac.jp; 6 Faculty of Human Sciences, Osaka University of Economics, Osaka 533-8533, Japan; eto@osaka-ue.ac.jp; 7 School of Nursing, The Jikei University, Tokyo 182-8570, Japan; jshimasawa@jikei.ac.jp (J.S.); yumiko_shimizu@jikei.ac.jp (Y.S.); 8 Center for Health Sciences and Counseling, Kyushu University, Fukuoka 819-0395, Japan; tsurugano@chc.kyushu-u.ac.jp; 9 Sports Medicine Research Center, Keio University, Yokohama 223-8521, Japan; fuminori@keio.jp; 10 Green Computing Systems Research Organization, Waseda University, Tokyo 169-8050, Japan; 11 Faculty of Science and Engineering, School of Fundamental Science and Engineering, Waseda University, Tokyo 169-8050, Japan; washizaki@waseda.jp; 12 Research Center for Realizing Sustainable Societies, The University of Electro-Communications, Tokyo 182-8585, Japan; ishigaki@uec.ac.jp; 13 Information Technology Center, The University of Tokyo, Chiba 277-8582, Japan; 14 Office for Research Management, The University of Electro-Communications, Tokyo 182-8585, Japan; suzuki.hitomi@uec.ac.jp

**Keywords:** health literacy, internet use, social relationship, older adults, cross-sectional study

## Abstract

**Background/Objectives**: Internet use positively impacts mental health in older adults, with health literacy (HL) playing a key role. While social networks may complement individual HL, the role of neighborhood relationships in this association, particularly by gender, remains unclear. This study examined how the association between HL and Internet use among older adults was modified by neighborhood relationships. **Methods**: Using baseline data from the Chofu–Digital–Choju project, a cross-sectional analysis was conducted on 1955 community-dwelling adults aged 65–84 (889 men and 1066 women). HL was assessed using the Communicative and Critical Health Literacy scale and dichotomized at four points. Neighborhood relationships were categorized as high (visiting/chatting with neighbors) or low (exchanging greetings/no relationship). Gender-stratified logistic regression analyses were performed with Internet use as the dependent variable, with HL, neighborhood relationships, and their interaction as independent variables. **Results**: Internet user proportion was 55.6% for men and 41.8% for women. HL was positively associated with Internet use in both genders, though patterns differed. Among men, the HL–Internet use association was consistent (OR = 3.09; 95% CI: 2.25–4.24) regardless of neighborhood relationship levels. For women, this association was significantly modified (interaction OR = 0.46, 95% CI: 0.24–0.87). Women with low HL but strong neighborhood relationships showed increased odds of Internet use (OR = 2.08, 95% CI: 1.32–3.26). **Conclusions**: Gender-specific patterns in HL and neighborhood relationships influence Internet use among older adults. Neighborhood relationships may compensate for low HL in women, underscoring the need for gender-sensitive strategies to promote digital HL.

## 1. Introduction

The digital divide among older adults remains a significant public health concern despite the global proliferation of Internet access. Although Internet usage is widespread, older adults lag behind other age groups in digital adoption and engagement [1]. Geographic disparities in Internet access are particularly pronounced between regions, with Asia, North America, and Europe having higher connectivity infrastructure compared to Africa [2], and these regional inequalities potentially influence economic development trajectories [3]. This pattern of regional digital divide extends to the national level—in Japan, significant technological disparities exist between urban and rural areas [4]. Besides geographic disparities, digital inequalities manifest between sociodemographic groups. In Japan, Internet usage among adults aged ≥60 years has increased by 7.5–10.8 percentage points from 2020 to 2023; however, non-users still outnumber those in younger age groups [5]. This digital disparity among older adults is consistently observed across various nations [6,7]. Recent evidence from a large-scale study across 23 countries demonstrated that Internet use was associated with fewer depressive symptoms, higher life satisfaction, and better self-reported health among older adults [8]. In Japan, Internet use—though not analyzed in the above study—has been associated with improved physical and cognitive health outcomes [9,10,11] and higher rates of health check-up participation [12]. These findings emphasize the importance of promoting Internet use in this population as a potential pathway to enhance well-being in later life.

Effectively utilizing online resources is intrinsically linked to health literacy (HL) [13,14], defined as “the cognitive and social skills which determine the motivation and ability of individuals to gain access to, understand and use information in ways which promote and maintain good health” [15]. Higher levels of HL are associated with reduced engagement in health risk behaviors such as smoking, regular alcohol consumption, and physical inactivity [16,17]. Among older adults, low HL contributes to frailty progression [18,19] and mortality [20]. Moreover, research has consistently demonstrated a significant correlation between HL and Internet use among older adults, with lower HL associated with a decreased likelihood of Internet engagement [13,21,22,23].

Evidence suggests that individual HL levels can be complemented by the skills and support of social networks [24,25]. This concept of “distributed health literacy” [25] emphasizes the potential for social relationships to compensate for individual limitations in health information processing and utilization. However, gender differences in social relationship patterns on the Internet suggest that the extent and nature of this complementary effect may vary between men and women [26,27]. Kim et al. indicate that men and women exhibit distinct Internet usage patterns, with women showing a greater tendency toward communication-oriented activities than men [26]. However, few studies have explored how gender differences in social relationships affect the link between HL and Internet use among older adults. Understanding these potential gender-specific pathways through which social relationships modify the HL–Internet use association could be crucial for developing targeted interventions.

To address this research gap, a cross-sectional study was conducted utilizing baseline data from a community-based intervention to examine how social relationships modify the association between HL and Internet use among older adults, with analyses conducted separately by gender. This investigation aims to elucidate the complex interplay between HL, social relationships, and Internet use, potentially informing more effective strategies for promoting digital HL among older adults.

## 2. Materials and Methods

### 2.1. Study Population

A cross-sectional analysis was conducted using baseline data from the “Chofu–Digital–Choju” (CDC; Choju means longevity in Japanese) project, a community-based intervention aimed at promoting health among community-dwelling older adults. The detailed methodology of this project has been previously described elsewhere [28,29]. In January 2022, a self-administered questionnaire with a unique identification number was mailed to 3742 individuals aged 65–84 living independently and registered with an address in two areas targeted by the project as of October 2021.

Informed consent was obtained through the questionnaire administered by the municipal government. While the municipality maintained the correspondence table linking personal identifiers to study data, researchers received only anonymized data after participants were given the opportunity to opt out. This procedure was approved by the University of Electro-Communications ethics committee (approval number: 21068).

### 2.2. Measures

#### 2.2.1. Internet Use

Internet use was assessed using a four-point scale measuring frequency: almost daily, 2–3 times per week, several times monthly, or no use. For analysis, Internet users are defined as those who reported almost daily use [30]. In this study, Internet use referred to routine activities such as browsing websites and sending or receiving emails [31,32].

#### 2.2.2. Health Literacy

HL as a dependent variable was measured using the Communicative and Critical Health Literacy (CCHL) scale, which directly reflects the World Health Organization’s definitions of communicative and critical literacy [33]. The scale comprises five items assessing advanced skills: (1) collecting health-related information, (2) extracting desired information, (3) understanding and communicating information, (4) evaluating information credibility, and (5) making decisions based on the information. Each item was rated on a 5-point scale (1 = strongly disagree to 5 = strongly agree), and the total score was calculated as the average of all items. Based on the previous research, participants were categorized into high and low HL groups using a cut-off score of four [33].

#### 2.2.3. Neighborhood Relationships

Neighborhood relationships were assessed by asking, “What kind of relationship do you have with people in your neighborhood?” [34,35]. The response options were: “visiting each other”, “standing and chatting”, “exchanging greetings”, and “none”. Based on a previous study, individuals who maintained active relationships with neighbors (i.e., those who reported “visiting each other” or “standing and chatting”) were classified as having high-level neighborhood relationships [34,35].

#### 2.2.4. Covariates

Social demographics included age, sex, financial status, employment status, smoking and drinking behaviors, body mass index, self-reported health status, depressive mood, and smartphone ownership. Age was categorized into four groups: 65–69, 70–74, 75–79, and 80–84 years. Financial status was evaluated using a self-rated 5-point Likert scale: “Low”, “Middle-low”, “Middle”, “Middle-high”, and “High”. [36]. Employment status was dichotomized as “currently employed” or “not employed.” Smoking and drinking status were each dichotomized as “current” or “former/never” users. Body mass index was calculated as self-reported weight (kg) divided by the square of self-reported height (m^2^) and categorized into three groups (<18.5, 18.5–24.9, or ≥25 kg/m^2^). These values were based on values within four standard deviations for age and sex [37], as reported in the National Health and Nutrition Survey in Japan [38]. Self-reported health status was assessed using a 4-point Likert scale ranging from “Poor” to “Excellent” and subsequently dichotomized into “excellent/good” or “fair/poor”. Depressive symptoms were evaluated using the five-item short form of the Geriatric Depression Scale (GDS-5; range 0–5), where a score of ≥2 indicated a depressive mood [39,40]. Smartphone ownership was assessed as part of regularly used devices but was excluded as a covariate in the analysis due to its role as an intermediate factor between HL and Internet use.

### 2.3. Statistical Analysis

Given our hypothesis regarding gender differences in social network utilization and their potential impact on digital behaviors, all analyses were stratified by sex. Descriptive statistics of participants’ characteristics were presented as numbers and percentages for categorical variables. The characteristics of Internet users and non-users were compared using chi-square tests stratified by sex. Then, we constructed four sequential logistic regression models to examine associations between HL, social relationships, and Internet use. Model 1 examined the unadjusted association between HL and Internet use. Model 2 incorporated HL and NRs as the main effects, adjusting for age. Model 3 applied full adjustment for potential confounders, whereas Model 4 introduced an interaction between HL and NRs with full adjustment. Moreover, to visualize the combined effects of HL and neighborhood relationships, we calculated odds ratios (ORs) for Internet use across four groups stratified by both factors (high/low HL × high/low NR) using logistic regression adjusted for all covariates. The group with low HL and neighborhood relationships was used as the reference category.

Spearman’s rank correlation coefficients were calculated to assess the validity of introducing these variables simultaneously. Consequently, we confirmed that no pairs of independent variables and covariates showed problematic correlations, with values ranging from −0.31 to 0.27. The assumption of linearity in the logit was addressed by dichotomizing variables (e.g., health literacy) using clinically meaningful cut-points established in previous research. Model fit was evaluated using Nagelkerke R^2^ and Hosmer–Lemeshow tests. Results were reported as odds ratios (ORs) with 95% confidence intervals (CIs). All analyses were conducted as complete case analyses and performed using IBM SPSS Statistics for Windows, version 29.0 (IBM Corp., Armonk, NY, USA), with statistical significance set at *p* < 0.05.

## 3. Results

**Figure 1:**
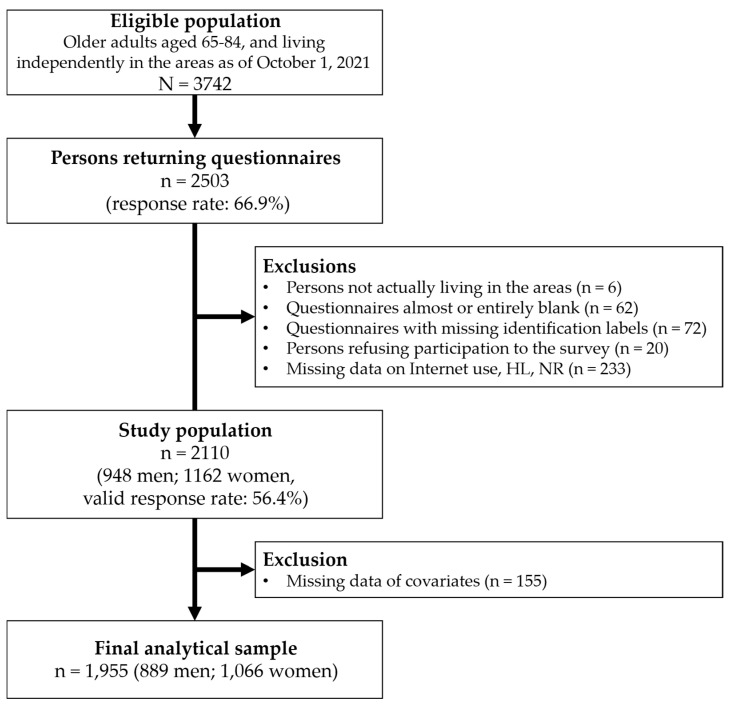
Study flowchart. HL: health literacy, NR: neighborhood relationship.

Of the 2503 respondents (response rate: 66.9%), data from 2110 participants (948 men and 1162 women; valid response rate: 56.4%) were analyzed after excluding 393 questionnaires. After excluding those with missing covariate data, the final analytical sample included 1955 participants (889 men and 1066 women; see Figure 1).

Of the 2110 eligible participants, 1955 with complete data for all variables were included in the main analysis (889 men and 1066 women)—Table 1 and Table 2 present characteristics of eligible participants, stratified by gender. Regular Internet use was reported by 527 men (55.6%) and 486 women (41.8%). Among men, Internet users were characterized by higher proportions of high HL (*p* < 0.001) and strong neighborhood relationships (*p* = 0.011) compared to non-users. A similar pattern was observed among women for HL (*p* < 0.001), but the difference in neighborhood relationships was less pronounced (*p* = 0.191). Internet use was significantly associated with age (*p* < 0.001) for both men and women. Compared to non-users, Internet users were more prevalent in the 65–69 and 70–74 age groups. Internet users of both genders were more likely to be employed (*p* = 0.002 for men; *p* = 0.005 for women) and to have a higher financial status (*p* < 0.001 for both sexes). Health-related behaviors and characteristics showed distinct patterns between Internet users and non-users. Among men, Internet users reported higher rates of current alcohol consumption (*p* < 0.001) and lower rates of current smoking (*p* = 0.015). Similar trends were observed among women, with Internet users showing higher rates of current alcohol consumption (*p* < 0.001). Although the association between Internet use and smoking status was marginally significant among women (*p* = 0.051), a trend towards lower smoking rates among Internet users was observed. Body mass index distributions differed significantly among men (*p* = 0.015), with Internet users showing a lower prevalence of underweight and similar rates of overweight. Among women, body mass index distributions were comparable between Internet users and non-users (*p* = 0.612). Among men, Internet users reported better self-rated health (*p* < 0.001) and a lower prevalence of depressive mood (*p* = 0.001). In women, only depressive mood was significantly associated with Internet use (*p* = 0.003). Internet users also showed significantly higher smartphone ownership rates in both genders (*p* < 0.001).

Table 3 illustrates the relationship between HL and NR with Internet use, as determined by logistic regression analysis. Among men, HL showed a strong and consistent relationship with Internet use across all models. In the age-adjusted model (Model 2), high HL was significantly associated with increased odds of Internet use (OR = 3.25, 95% CI: 2.40–4.39, *p* < 0.001). This association remained robust in the fully adjusted model (Model 3), accounting for potential confounders (OR = 3.09, 95% CI: 2.25–4.24, *p* < 0.001). In Model 4, the introduction of an interaction term between HL and neighborhood relationships revealed no significant modification effect in men (OR = 1.18, 95% CI: 0.64–2.19, *p* = 0.590), indicating that the association between HL and Internet use was independent of neighborhood relationship levels. Among women, the associations followed a more complex pattern. In the age-adjusted model (Model 2), high HL was significantly associated with increased odds of Internet use (OR = 2.55, 95% CI: 1.96–3.31, *p* < 0.001), and this association persisted in the fully adjusted model (Model 3; OR = 2.28, 95% CI: 1.74–2.99, *p* < 0.001). However, in Model 4, the addition of the interaction term revealed a significant modification effect by neighborhood relationships (interaction OR = 0.46, 95% CI: 0.24–0.87, *p* = 0.017). Notably, the association between HL and Internet use was markedly stronger among women with low neighborhood relationships (OR = 4.10, 95% CI: 2.34–7.17, *p* < 0.001).

**Figure 2:**
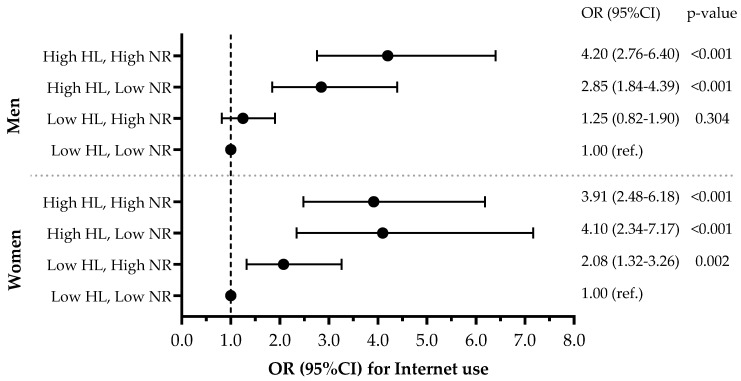
Odds ratio of Internet use by the levels of combination between HL and NR. Analyses were adjusted for age, alcohol consumption, smoking, employment status, financial status, body mass index, health status, and depressive mood. OR: odds ratio, CI: confidential interval, HL: health literacy, NR: neighborhood relationship.

Figure 2 presents the ORs for Internet use across different combinations of HL and NR, with the low HL/low NR group as the reference category. Among men, high HL was strongly associated with Internet use, regardless of NR status (high HL/high NR: OR = 4.20, 95% CI: 2.76–6.40; high HL/low NR: OR = 2.85, 95% CI: 1.84–4.39). Among women, high HL showed strong associations with Internet use across NR levels (high HL/high NR: OR = 3.91, 95% CI: 2.48–6.18; high HL/low NR: OR = 4.10, 95% CI: 2.34–7.17). Notably, even women with low HL but high NR demonstrated significantly higher odds of Internet use (OR = 2.08, 95% CI: 1.32–3.26, *p* = 0.002).

Additional analyses of other forms of social relationships (social participation, social isolation, and living arrangement) revealed no significant interaction effects with HL for either gender (results not displayed).

## 4. Discussion

This study reveals distinct gender patterns in how HL and neighborhood relationships interact to influence Internet use among older adults. Our findings demonstrate that while HL is consistently associated with Internet use across both genders, the moderating role of social relationships differs markedly between men and women, suggesting complex gender-specific pathways in digital engagement among older adults. These findings contribute to understanding the intricate relationships between individual capabilities, social context, and digital engagement in later life.

The association between HL and Internet use observed in both men and women aligns with previous research documenting the crucial role of HL in digital engagement [13,21,22,23]. However, our study extends these findings by revealing gender-specific patterns in how social relationships moderate this relationship. Among men, the relationship between HL and Internet use remained robust and independent of neighborhood relationship levels, suggesting that men’s digital engagement may be more individually driven. This finding aligns with previous research indicating that men use information and communication technology less for social purposes than women [26,27,41]. One of our most striking findings is the significant interaction effect between HL and neighborhood relationships, observed exclusively among women. These findings support the distributed HL framework [25,42], particularly among women. Women with strong neighborhood relationships showed a weaker association between HL and Internet use than those with weak neighborhood relationships, suggesting that social relationships may complement individual HL skills. This pattern aligns with the concept that HL can be distributed across social networks, potentially reducing the reliance on individual HL capabilities when strong social relationships exist.

This gender-specific pattern can be further understood through the frameworks of distributed HL and the complementary hypothesis. The complementary hypothesis suggests that individuals with existing social connections are more likely to use the Internet to strengthen their existing networks [41,43]. This is consistent with previous findings indicating that individuals living in neighborhoods with higher Internet usage rates are more likely to engage in online communication [44,45,46,47]. In our study, women with strong neighborhood relationships demonstrated a weaker association between individual HL and Internet use, suggesting they may leverage social networks for HL resources and Internet adoption. Conversely, women with limited neighborhood relationships, like men, showed a stronger reliance on individual HL for Internet engagement, indicating that personal capabilities become more crucial without strong social networks. Additionally, the unique role of neighborhood relationships in moderating the HL–Internet use association is particularly noteworthy. Additional analyses examining other forms of social relationships (social participation, social isolation, and living arrangement) revealed no significant interaction effects with HL for either gender. This suggests that the moderating effect on the HL–Internet use relationship might be specific to neighborhood relationships.

This study provides novel insights into the gender-specific pathways through which HL and neighborhood relationships influence Internet use among older adults. Our findings reveal that while HL is consistently associated with Internet use across both genders, the role of neighborhood relationships differs markedly between men and women. For women, neighborhood relationships significantly moderate the association between HL and Internet use, suggesting that social context is crucial in their digital engagement. In contrast, men’s Internet use appears to be more individually driven, with HL maintaining a consistent effect regardless of social relationships. These findings have important implications for both theory and practice. They highlight the need for gender-sensitive approaches in promoting digital HL among older adults, considering both individual capabilities and social context. For women with limited neighborhood relationships, interventions should focus on enhancing individual HL skills while simultaneously fostering new social connections. For those with strong neighborhood relationships, leveraging existing social networks may be more effective.

However, several limitations should be considered when interpreting our findings. First, the cross-sectional design of our study precludes causal inference regarding the relationships between HL, neighborhood relationships, and Internet use. Longitudinal studies are needed to establish temporal relationships and examine how changes in social relationships influence digital engagement over time. Second, our response rate (66.9%) was relatively low compared to similar surveys of community-dwelling older adults [48,49], which may affect the representativeness of our sample. Additionally, our complete case analysis approach reduced the analytical sample to 1955 participants due to missing data, which may have introduced selection bias. Future studies should consider using multiple imputation techniques to handle missing data more efficiently. Third, our reliance on self-reported measures for Internet use and HL may have introduced reporting bias, although we used validated scales to minimize this concern. Fourth, this study was conducted in an urban Japanese setting, and the findings may not be fully generalizable to other cultural contexts or rural areas, where patterns of neighborhood relationships and Internet use may differ. Moreover, a notable limitation is the lack of data on participants’ educational attainment. Educational background in Japan shows regional variations [50], with men generally having greater access to higher education compared to women [50,51]. Given that previous research has demonstrated associations between Internet use and both educational level and social relationships [52,53], our findings could be potentially confounded by this unmeasured variable. Finally, while we measured the presence of neighborhood relationships, we did not assess their quality or specific nature, which may provide additional insights into how these relationships influence digital engagement.

Future research should address several key areas. First, longitudinal studies are needed to establish causal relationships and examine how changes in social relationships influence digital engagement over time. Second, qualitative research could provide deeper insights into the specific mechanisms through which neighborhood relationships influence technology adoption, particularly among women. Third, cross-cultural studies would be valuable in understanding how these relationships manifest across different societal contexts, where gender roles and neighborhood relationships may vary. Finally, intervention studies testing gender-sensitive, community-based approaches to promoting digital HL would help translate our findings into practical applications.

## 5. Conclusions

Our findings reveal that while HL is consistently associated with Internet use across both genders, the role of neighborhood relationships differs markedly between men and women. For women, neighborhood relationships significantly moderate the association between HL and Internet use, suggesting that social context is crucial in their digital engagement. In contrast, men’s Internet use appears to be more individually driven, with HL maintaining a consistent effect regardless of social relationships.

Future interventions should consider these gender differences when designing strategies to promote Internet use. For men, programs focusing on enhancing individual HL may be relevant, while for women, particularly those with strong neighborhood relationships, approaches that incorporate existing social networks could be considered alongside HL enhancement. Such targeted approaches could maximize the adoption and benefits of Internet use among older adults, potentially contributing to better health outcomes in this population.

## Figures and Tables

**Table 1:** Participation characteristics among men (n = 948).

	Non-User, n = 421, 44.4%		Internet User, n = 527, 55.6%		
Variables	n	%	n	%	*p*-value
Health literacy					<0.001
Low	280	66.5	206	39.1	
High	141	33.5	321	60.9	
Neighborhood					0.011
No	237	56.3	253	48.0	
Yes	184	43.7	274	52.0	
Age					<0.001
65–69	57	13.5	177	33.6	
70–74	110	26.1	189	35.9	
75–79	129	30.6	85	16.1	
80–84	125	29.7	76	14.4	
Alcohol					<0.001
Never or Former	138	32.9	113	21.4	
Current	282	67.1	414	78.6	
Smoking					0.015
Never or Former	340	81.1	458	86.9	
Current	79	18.9	69	13.1	
Employment status					0.002
No	260	62.2	273	51.9	
Yes	158	37.8	253	48.1	
Financial status					<0.001
Low	20	4.8	19	3.6	
Middle–low	71	17.1	54	10.3	
Middle	179	43.0	158	30.1	
Middle–high	131	31.5	260	49.5	
High	15	3.6	34	6.5	
Body mass index, kg/m^2^					0.015
<18.5	22	5.4	10	1.9	
18.5–24.9	286	69.9	382	73.2	
≥25	101	24.7	130	24.9	
Self-rated health					<0.001
Fair to poor	101	24.4	73	14.0	
Excellent to good	313	75.6	450	86.0	
Depressive mood					0.001
Absence	232	57.4	353	67.8	
Presence	172	42.6	168	32.2	
Owning smartphone					<0.001
No	233	55.7	74	14.0	
Yes	185	44.3	453	86.0	

Missing values were removed. *p*-values were calculated using the chi-square test to compare Internet users and non-users.

**Table 2:** Participation characteristics among women (n = 1162).

	Non-User, n = 676, 58.2%		Internet User, n = 486, 41.8%		
Variables	n	%	n	%	*p*-value
Health literacy					<0.001
Low	432	63.9	194	39.9	
High	244	36.1	292	60.1	
Neighborhood					0.191
No	180	26.6	113	23.3	
Yes	496	73.4	373	76.7	
Age					<0.001
65–69	88	13.0	155	31.9	
70–74	202	29.9	166	34.2	
75–79	212	31.4	115	23.7	
80–84	174	25.7	50	10.3	
Alcohol					<0.001
Never or Former	425	63.2	227	46.9	
Current	248	36.8	257	53.1	
Smoking					0.051
Never or Former	634	94.2	467	96.7	
Current	39	5.8	16	3.3	
Employment status					0.005
No	512	77.5	338	70.1	
Yes	149	22.5	144	29.9	
Financial status					<0.001
Low	17	2.5	15	3.1	
Middle–low	95	14.2	36	7.4	
Middle	298	44.5	161	33.3	
Middle–high	228	34.0	248	51.2	
High	32	4.8	24	5.0	
Body mass index, kg/m^2^					0.612
<18.5	71	10.9	47	9.8	
18.5–24.9	459	70.3	351	73.0	
≥25	123	18.8	83	17.3	
Self-rated health					0.107
Fair to poor	105	15.6	59	12.3	
Excellent to good	567	84.4	422	87.7	
Depressive mood					0.003
Absence	407	62.0	333	70.6	
Presence	249	38.0	139	29.4	
Owning smartphone					<0.001
No	331	49.6	43	8.8	
Yes	337	50.4	443	91.2	

Missing values were removed. *p*-values were calculated using the chi-square test to compare Internet users and non-users.

**Table 3:** Gender-specific associations between health literacy, neighborhood relationships, and Internet use.

	Model 1		Model 2		Model 3		Model 4	
	Crude		Age-adjusted		Multivariable-adjusted *		Interaction term included ^†^	
	Odds ratio (95% CI)	*p*-value	Odds ratio (95% CI)	*p*-value	Odds ratio (95% CI)	*p*-value	Odds ratio (95% CI)	*p*-value
**Men**, n = 889								
Health literacy (ref = low)	3.16 (2.40–4.18)	<0.001	3.25 (2.40–4.39)	<0.001	3.09(2.25–4.24)	<0.001	2.85 (1.84–4.39)	<0.001
Neighborhood (ref = low)			1.46 (1.08–1.98)	0.013	1.35 (0.99–1.84)	0.061	1.25 (0.82–1.90)	0.304
Health literacy × Neighborhood							1.18 (0.64–2.19)	0.590
**Model fit** (Nagelkerke R^2^/Hosmer–Lemeshow test)	0.100/-		0.235/0.482		0.284/0.202		0.284/0.586	
**Women**, n = 1066								
Health literacy (ref = low)	2.64 (2.05–3.39)	<0.001	2.55 (1.96–3.31)	<0.001	2.28 (1.74–2.99)	<0.001	4.10 (2.34–7.17)	<0.001
Neighborhood (ref = low)			1.45 (1.06–1.97)	0.020	1.43 (1.04–1.97)	0.028	2.08 (1.32–3.26)	0.002
Health literacy × Neighborhood							0.46 (0.24–0.87)	0.017
**Model fit** (Nagelkerke R^2^/Hosmer–Lemeshow test)	0.073/-		0.178/0.794		0.217/0.398		0.223/0.334	

* Additionally, adjustments were made for drinking, smoking, employment, financial status, body mass index, overall health status, and depressive mood. ^†^ An interaction term was introduced with all of the previous covariates.

## Data Availability

No new data were created or analyzed in this study. Data sharing is not applicable to this article.
